# The Medical Superintendent

**Published:** 1905-04-01

**Authors:** 

**Affiliations:** Chef du service d'hygiène de Schaerbeek, 37 Rue Royale Ste. Marie, Bruxelles


### EDITOR'S LETTER-BOX.

**[Our Correspondents are reminded that prolixity is a great bar to publication, and that brevity of style and conciseness of statement greatly facilitate early insertion.]**

### THE MEDICAL SUPERINTENDENT.

Sir,—I have read with much interest your article on the Medical Superintendent, in your "Hospitals and Charities." May I apply to you for following question? Do you know some publication where I could find the salaries paid to medical superintendents and medical officers in hospitals asylums? You should oblige me very much, if you could give me some information on that point.

Yours faithfully,
Dr. Eusch,
Chef du service d'hygiène de Schaerbeek,
37 Rue Royale Ste. Marie, Bruxelles.

